# Image quality & dosimetric property of an investigational Imaging Beam Line MV‐CBCT

**DOI:** 10.1120/jacmp.v10i3.3023

**Published:** 2009-06-17

**Authors:** Chris Beltran, Renin Lukose, Bijumon Gangadharan, Ali Bani‐Hashemi, Bruce A. Faddegon

**Affiliations:** ^1^ Department of Radiological Sciences St. Jude Children's Research Hospital Memphis TN USA; ^2^ Siemens Medical USA Concord CA USA; ^3^ Department of Radiation Oncology University of California San Francisco San Francisco CA USA

**Keywords:** MV‐CBCT, image quality, imaging beam line, dosimetric comparison

## Abstract

To measure and compare the contrast to noise ratio (CNR) as a function of dose for the CBCTs produced by the mega‐voltage (MV) imaging beam line (IBL) and the treatment beam line (TBL), and to compare the dose to target and various critical structures of pediatric patients for the IBL CBCT versus standard TBL orthogonal port films.

Two Siemens Oncor linear accelerators were modified at our institution such that the MV‐CBCT would operate under an investigational IBL rather than the standard 6MV TBL. Prior to the modification, several CBCTs of an electron density phantom were acquired with the TBL at various dose values. After the modification, another set of CBCTs of the electron density phantom were acquired for various doses using the IBL. The contrast to noise ratio (CNR) for each tissue equivalent insert was calculated. In addition, a dosimetric study of pediatric patients was conducted comparing the 1 cGy IBL CBCT and conventional TBL orthogonal pair port films.

The CNR for eight tissue equivalent inserts at five different dose settings for each type of CBCT was measured. The CNR of the muscle insert was 0.8 for a 5 cGy TBL CBCT, 1.1 for a 1.5 cGy IBL CBCT, and 2.8 for a conventional CT. The CNR of the trabecular bone insert was 2.9 for a 5 cGy TBL CBCT, 5.5 for a 1.5 cGy IBL CBCT, and 14.8 for a conventional CT. The IBL CBCT delivered approximately one‐fourth the dose to the target and critical structures of the patients as compared to the TBL orthogonal pair port films.

The IBL CBCT improves image quality while simultaneously reducing the dose to the patient as compared to the TBL CBCT. A 1 cGy IBL CBCT, which is used for bony anatomy localization, delivers one‐fourth the dose as compared to conventional ortho‐pair films.

PACS number: 87.57.Q, 87.57.cj, 87.53.Jw

## I. INTRODUCTION

Cone Beam Computed Tomography^(1)^ (CBCT) is routinely used for treatment localization based on anatomical structures or implanted markers for a variety of sites.^(^ ^2^ ^–^ ^11^ ^)^ The CBCT is a volumetric dataset; therefore, it can be directly compared and registered to the planning simulation CT, giving more information than traditional orthogonal planar images. Currently, there exist three commercially available CBCT systems: the Varian On‐Board‐Imaging (OBI) (Varian Medical Systems, Palo Alto, CA), the Elekta XVI Synergy system (Elekta, Stockholm, Sweden), and the Siemens MVision system (Siemens Medical Solutions, Malvern, PA). The first two are kilovoltage (kV) systems, which require a kV source and imaging panel mounted at right angles from the treatment beam; the third system is a megavoltage (MV) system that does not require additional hardware components.^(12)^

In light of ALARA, the Image Gently Campaign, and the increased risk of secondary malignancies for pediatric patients^(13)^ resulting from relatively low radiation doses, it is crucial to minimize the localization imaging dose while simultaneously ensuring accurate and precise setup. The need to have reliable, efficient, and effective localization has become increasingly clear as high dose conformal radiation therapy, including intensity modulated radiation therapy (IMRT)^(14)^ and proton therapy^(15)^ enter the mainstream for children. Recently, Faddagon et al.^(^ ^16^ ^,^ ^17^ ^)^ introduced a modification to the Siemens MV‐CBCT (referred to as the imaging beam line (IBL)) to allow for improved image quality at a lower radiation dose as compared to the treatment beam line (TBL).

In this paper, we compare the contrast to noise ratio (CNR) as a function of dose for the MV‐CBCTs produced by the IBL and the TBL. In addition, we compare the dose to the target and to various critical structures of pediatric patients for the IBL CBCT versus the standard of care orthogonal pair port films (ortho‐pair). This work was undertaken for the primary purpose of establishing the efficacy of this investigational system for an institutional review board (IRB) protocol in which all 3D‐CRT/IMRT pediatric patients will undergo daily localization with IBL‐CBCT.^(11)^

## II. MATERIALS AND METHODS

### A. CBCT image quality: IBL vs. TBL

Two Siemens Oncor linear accelerators with MVision (Siemens Medical Solutions, Malvern, PA) were modified at our institution such that the MV‐CBCT would operate under an investigational IBL^(16)^ rather than the standard 6 MV TBL. The major modifications consisted of replacing the tungsten target with a carbon target, removing the fattening filter, and decreasing the beam energy by 30% to 4.2 MeV, resulting in a photon beam with a mean energy of approximately 800 keV as opposed to the standard mean energy of approximately 2 MeV for the TBL. The IBL replaced the 18–21 MeV electron treatment beam line. The electron ion chambers were used for the IBL and were calibrated such that 3 MU is 1 cGy at $d_{\max}$ (1 cm depth, 100 SSD). The details of the modifications are explained in depth by Faddagon et al.^(16)^ Depth dose, profiles, and output for various field sizes of the IBL and TBL were measured and modeled in the PlanUNC (University of North Carolina, Chapel Hill NC) treatment planning system (TPS). Dose calculations from the TPS were verified with ion chamber measurements. Daily image quality and twice‐weekly output and energy checks are routinely performed on the IBL system to ensure proper functionality.

Prior to the modification, several CBCTs of an electron density phantom (CIRS model 062) were acquired with the TBL at various “dose to isocenter” values, where the isocenter was set to the center of the phantom. The phantom had an average diameter of 33 cm, with two concentric rings of tissue equivalent inserts. The inner ring of inserts was at a radius of 11.5 cm. The various tissue equivalent inserts and their densities are listed in Table 1. After the machine modification, another set of CBCTs of the electron density phantom were acquired at various “dose to isocenter” values using the IBL. A simulation CT was also acquired using a Siemens SOMATOM CT. The reconstruction slice width was set to 5 mm for all images.

**Table 1:** The various tissue equivalent inserts and densities of the electron density phantom.

*Low Contrast Material*		*High Contrast Material*	
*Tissue Equivalent Inserts*	*Density Relative to Water*	*Tissue Equivalent Inserts*	*Density Relative to Water*
Breast	−1%	Lung Inhale	−80%
Adipose	−3%	Lung Exhale	−50%
Muscle	+6%	Trabecular Bone	+16%
Liver	+7%	Dense Bone	+61%

**Figure 1:**
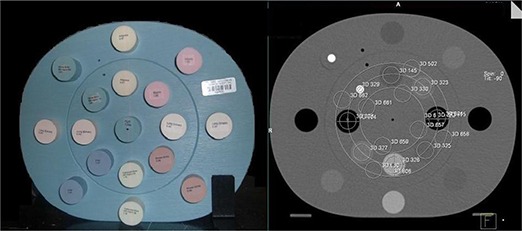
Image (left) and CT (right) of the heterogeneity phantom. The regions of interests shown on the CT are the same that are used for the various CBCTs.

For each CBCT, a region of interest (ROI) of 4.5 cm^2^ was outlined for each of the different electron density inserts in the inner ring of the phantom and for the water equivalent material near each insert. The ROI for the dense bone was smaller because of the insert size for that material. Figure 1 shows the image (left) and CT (right) of the heterogeneity phantom. The regions of interests shown on the CT are the same as those used for the various CBCTs. The 3D module within the Siemens Coherence system was used to gather the data. This module allows one to draw ROIs and gives pertinent statistics about that region. The mean pixel value (Signal) and standard deviation of the pixel values (Noise) for each region of interests were recorded. The contrast to noise ratio (CNR) for each tissue equivalent area was calculated based on the following; ${\text{CNR}}_{\text{tissue}}=C_{\text{tissue}}/N_{\max}$, where $N_{\max}=(\text{Noise}\;\text{of}\;\text{Tissue}\;\text{equivalent}\;\text{or}\;\text{Noise}\;\text{of}\;\text{nearest}\;\text{Water}\;\text{equivalent})$, and $C_{\text{tissue}}=[(\text{Signal}\;\text{of}\;\text{Tissue}\;\text{equivalent})-(\text{Signal}\;\text{of}\;\text{nearest}\;\text{Water}\;\text{equivalent})]\times \text{sign}[(\text{Density}\;\text{of}\;\text{Tissue}\;\text{equivalent})-(\text{Density}\;\text{of}\;\text{Water})]$. If the contrast as defined above was a negative value, it was set to zero.

### B. Dosimetric comparison between IBL CBCT and TBL orthogonal port films

A dosimetric study of the first 33 research participants on the Institutional Review Board (IRB) approved IBL protocol^(11)^ was conducted comparing the investigational IBL CBCT and TBL ortho‐pair. Each CBCT delivered 1cGy to the treatment isocenter. Per the IRB protocol, the CBCT was to be done pretreatment daily and post‐treatment every other day. Based on phantom studies, a dose of 1 cGy was deemed sufficient to distinguish bony anatomy. Therefore, 1 cGy was chosen to keep the imaging dose as low as reasonably achievable. The patients were broken up into two cohorts, cranial and body. Cranial patients included patients with targets in the head and neck region. Body patients included all other sites including chest, abdomen, and pelvis. Patients with targets in the extremities were not included in this dosimetric study because none had been accrued at the time (although extremity sites were eligible for the protocol).

#### B.1. Beam setup

CBCT planning used the IBL beam modeled in the PlanUNC TPS as previously described. The planning technique was the same as the delivery, which was a 200° arc starting from 270° (which is a right lateral for a head first supine patient) to 110° with one projection per degree. The dose distribution is the summation of dose from 200 subfields. The field size was defined transversely to cover the whole cranium or body and longitudinally by the target volumes and critical organs. Critical organs were spared wherever possible by limiting the longitudinal field size. For this dose comparison study, the planned CBCT dose was set to 1.1 cGy, giving a worst‐case scenario that may result from rounding of the monitor units (MU).

The ortho‐pair planning used the 6 MV TBL beam data modeled in the PlanUNC TPS. The technique was an anterior‐posterior (AP) and a right‐lateral (Lat) field. The field size was set per our current clinical practice, which is to set the field size such that it extends 5 cm to 7 cm beyond the treatment field edges. The beam on time was a total of 4 MU (2 MU‐AP, 2 MU‐Lat) for cranial patients, and 5 MU (2 MU‐AP, 3 MU‐Lat) for body patients.

#### B.2. Dosimetric comparison

To take into account the non‐uniform dose distribution and their radiobiological equivalence, the quantitative dose comparison was done using Niemierko's generalized equivalent uniform dose (gEUD) formula^(18)^. The *a* value parameter of the gEUD equation was selected based on the radiosensitivity of the organs and target.^(19)^ For the target volumes, the *a* value was set to −10. For serial type critical organs, the *a* value was set to 10, and it was set to 2 for parallel type critical organs. In addition, the isocenter dose, mean dose, volume‐receiving 0.1cGy, and the maximum dose (defined as the dose to the hottest 3% of the entire volume) were obtained.

Further critical volume dose reductions were studied for two teenaged female body patients with targets on their left side. An IBL treatment plan with the CBCT arc starting at 340° to 180° (a left arc as opposed to the standard anterior arc) was created in order to investigate dose reduction to the contra‐lateral breast.

## III. RESULTS

### A. CBCT image quality: IBL vs. TBL

**Figure 2:**
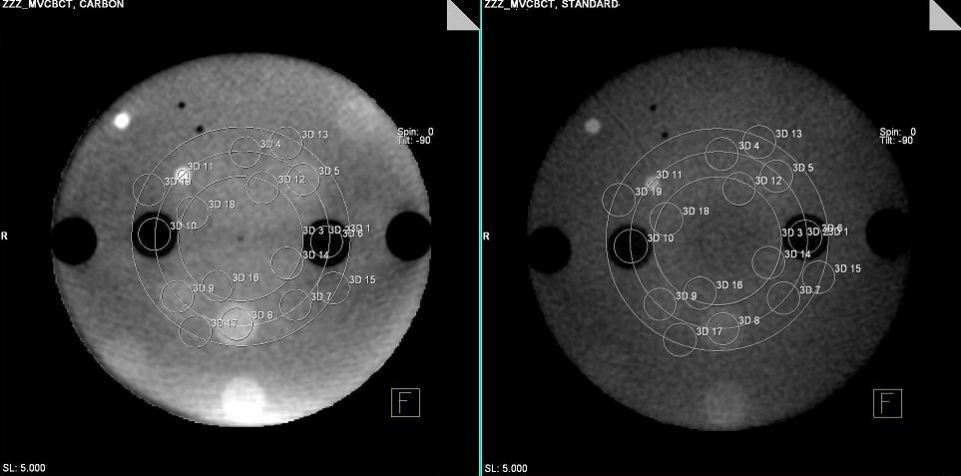
The center slice of the heterogeneity phantom with corresponding regions of interests for a 2 cGy IBL CBCT (left) and a 5 cGy TBL CBCT (right).

**Figure 3:**
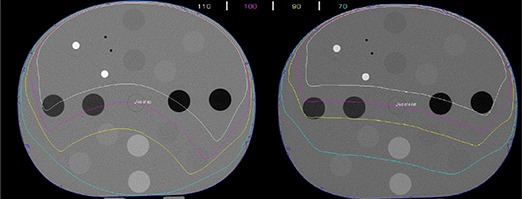
The relative dose distribution for the TBL (left) and IBL (right) CBCT. The 100% line intersects the isocenter.

**Figure 4:**
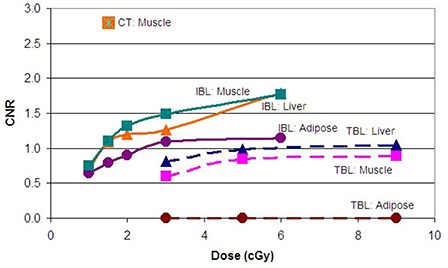
Graph of the CNR for the low‐contrast medium in the phantom for the Imaging Beam Line (solid) and Treatment Beam Line (dashed) CBCTs.

**Figure 5:**
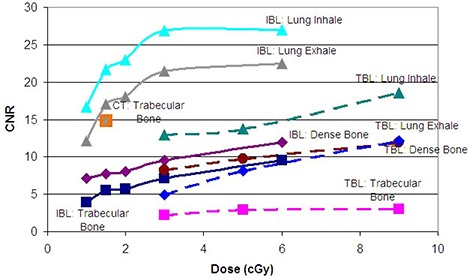
Graph of the CNR for the high‐contrast medium in the phantom for the Imaging Beam Line (solid) and Treatment Beam Line (dashed) CBCTs.

The minimum isocenter dose the system can reasonably deliver to this phantom was 1 cGy for the IBL and 3 cGy for the TBL. Therefore, the following doses were examined in detail: 1, 1.5, 2, 3 and 6 cGy for the IBL; 3, 5, 9 and 36 cGy for the TBL. Figure 2 is the center slice of the heterogeneity phantom for a 2 cGy IBL CBCT (left) and a 5 cGy TBL CBCT (right). Figure 3 shows the relative dose distribution for the IBL and TBL CBCT. Figure 4 is a graph of the CNR for the low‐contrast medium in the phantom. Note that the breast insert is not graphed, as the CNR was 0 for all CBCTs. In addition, the 36 cGy values for the TBL CBCT are not shown, as the increase in CNR was <10% of the 9 cGy values. Figure 5 is a graph of the CNR of the high‐contrast medium. In addition, the CT CNR for muscle (low contrast) and trabecular bone (high contrast) are shown. The estimated dose for the CT was 1.5 cGy.

### B. Dosimetric comparison between IBL CBCT and TBL orthogonal port films

**Figure 6:**
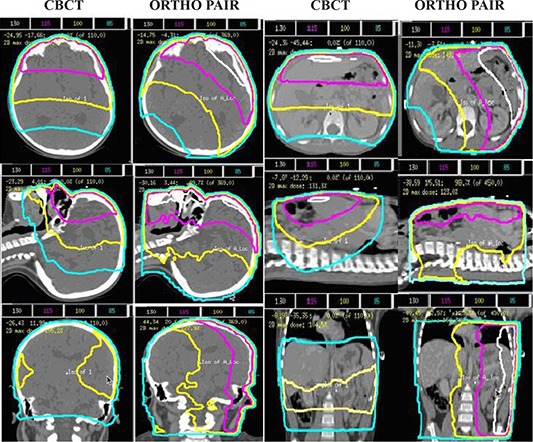
The dose distributions, normalized to isocenter, on transverse, coronal and sagittal planes along the isocenter of a cranial and a body patient for an IBL CBCT and ortho‐pair plan.

Data form 33 patients (15 males, 18 females) with an average age of 9.4 (range 1–25) years was used in this study. Figure 6 shows the dose distributions, normalized to isocenter on transverse, coronal and sagittal planes along the isocenter of a cranial and a body patient for an IBL CBCT and ortho‐pair plan. For the CBCT, because of anterior arc, there is uniformity along lateral direction and dose gradient along anterior posterior direction. This leads to more dose to anterior critical organs. In ortho‐pair imaging, the high‐dose area is located where the orthogonal beams intersect leading to a dose difference to the bilateral organs. The imaging field sizes ranged form $27\times 13\;{\text{cm}}^{2}$ to $27\times 26\;{\text{cm}}^{2}$ for the CBCTs, and $14\times 14\;{\text{cm}}^{2}$ to $30\times 30\;{\text{cm}}^{2}$ for the ortho‐pairs.

**Figure 7:**
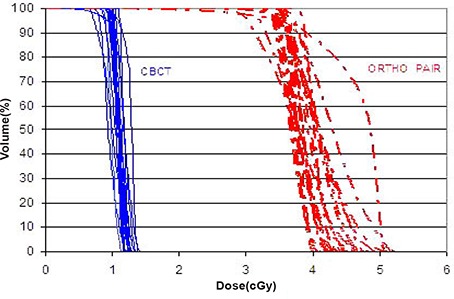
The CBCT (solid) and ortho‐pair (dashed) Dose Volume Histograms (DVH) for the clinical target volume (CTV) of the 23 cranial patients.

**Figure 8:**
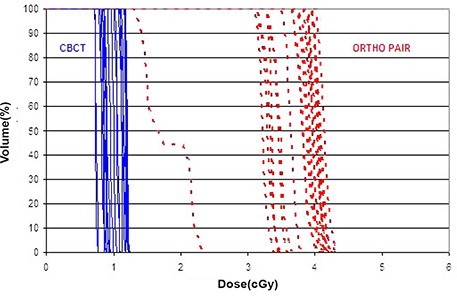
The CBCT (solid) and ortho‐pair (dashed) Dose Volume Histograms (DVH) for the optic chiasm of the 23 cranial patients.

**Figure 9:**
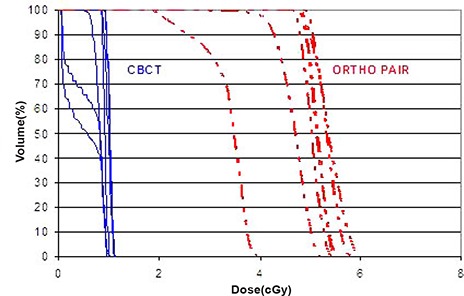
The CBCT (solid) and ortho‐pair (dashed) Dose Volume Histograms (DVH) for the left kidney of the 10 body patients.

For this study, the dose calculated for each patient in the TPS was for 10 CBCTs or 10 ortho‐pairs and the dose reported here was divided by 10 to give the dose per CBCT or ortho‐pair. Measurements at two points within a cubic solid water phantom, central axis 5 cm deep and 5 cm off‐axis, were made to compare the TPS predicted dose and measured dose. The results were within 2% on central axis and 4% off‐axis. The Dose Volume Histograms (DVH) of various critical organs and target volumes from CBCT and ortho‐pair are shown in Figs. 7, 8, and 9. Table 2 shows the mean ±1 standard deviation of the gEUD values for target volumes and the critical organs from the CBCT and ortho‐pair for 23 cranium patients, and Table 3 contains the values for the 10 body patients. In addition, the isocenter dose, mean dose, volume receiving 0.1 cGy, and the maximum dose are included.

**Table 2:** The mean ±1 standard deviation of the gEUD values for target volumes and the critical organs from the CBCT and ortho‐pair for 23 cranium patients. The isocenter dose, mean dose, volume receiving 0.1 cGy, and the maximum dose are also included.

*Volume*	*gEUD CBCT (cGy*)	*gEUD ORTHO‐PAIR (cGy*)
CTV	1.03±0.13	3.68±0.60
GTV	1.07±0.10	3.80±0.43
PTV	0.96±0.24	3.31±1.04
Cord	0.79±0.21	3.58±0.35
Optic Nerve (right)	1.07±0.24	3.85±0.54
Optic Nerve (left)	1.07±0.24	3.99±0.75
Optic Chiasm	1.01±0.16	3.73±0.48
Lens (right)	1.03±0.29	3.43±0.90
Lens (left)	1.04±0.29	3.71±1.05
Cochlea (right)	0.97±0.12	3.61±0.26
Cochlea (left)	0.97±0.13	3.85±0.40
Isocenter	1.10±0.00	3.81±0.14
3%Vol Dose	1.23±0.08	4.65±0.20
Mean Dose	0.47±0.13	2.23±0.67
Volume Receiving 0.1cGy	3155.8±1017.1cc	6811.4±8210.6cc

**Table 3:** The mean ±1 standard deviation of the gEUD values for target volumes and the critical organs from the CBCT and ortho‐pair for 10 body patients. The isocenter dose, mean dose, volume receiving 0.1 cGy, and the maximum dose are also included.

*Volume*	*gEUD CBCT(cGy*)	*gEUD ORTHO‐PAIR (cGy*)
CTV	0.99±0.08	4.48±0.25
GTV	1.02±0.08	4.55±0.32
PTV	0.91±0.12	3.99±1.33
Cord	0.89±0.05	4.11±0.43
Heart	0.88±0.30	4.34±1.30
Liver	1.03±0.12	4.09±0.69
Lung (right)	0.71±0.22	3.26±1.11
Lung (left)	0.76±0.18	3.79±0.86
Kidney (right)	0.84±0.19	3.99±0.26
Kidney (left)	0.87±0.16	4.86±0.70
Spleen	0.64±0.00	4.18±0.00
Thyroid	1.21±0.00	4.58±0.00
Breast (right)	0.42±0.11	1.09±0.19
Breast (left)	0.63±0.10	3.60±0.44
Bladder	1.13±0.00	3.77±0.00
Rectum	0.74±0.00	3.03±0.00
Isocenter	1.10±0.00	4.50±0.19
3% Vol Dose	1.25±0.08	5.89±0.33
Mean Dose	0.39±0.09	2.12±0.58
Volume Receiving 0.1cGy	7935.9±4807.5cc	13285.5±7822.7cc

Replanning of the two teenaged female patients showed that the average dose to the contra‐lateral breast could be reduced from 0.42 cGy to 0.17 cGy per IBL CBCT.

## IV. DISCUSSION

**Figure 10:**
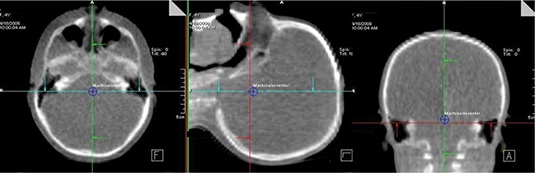
An image of a 1 cGy IBL CBCT for a 4‐year‐old female patient.

The IBL CBCT does improve image quality while simultaneously decreasing the dose required to achieve the quality as compared to the TBL CBCT. For comparable image quality, approximately one‐fourth the dose is required for the IBL. Using a different phantom, Faddegon et al.^(16)^ showed a similar trend in dose versus image quality for both the IBL and TBL. Thus, the IBL can be installed in different institutions and maintain dosimetric and image quality improvements. Gayou et al.^(20)^ measured similar CNR versus dose for the TBL CBCT. At 1 cGy per CBCT, an image of sufficient contrast can be obtained for localization based on bone anatomy for cranial and body patients. This is illustrated in Fig. 10, which is an image of a 1 cGy IBL CBCT for a 4‐year‐old patient. This low‐dose imaging is made possible primarily by the abundance of low energy photons. These photons have the advantage of better image quality with a lower dose to the patient due to higher quantum efficiency of the detector and greater contrast for different tissue types in the low energy range.^(16)^

An interesting observation is that while the liver equivalent insert has a higher density than the muscle insert (1.07 vs. 1.06), its CNR is lower: 1.2 vs. 1.3 for 2 cGy. This is due to the anterior hemispherical arc of the CBCT and the fact that the muscle insert was placed in the anterior portion of the phantom and the liver insert in the posterior portion. A repeat 2 cGy IBL CBCT was performed with the phantom flipped, and the resulting CNR was higher for the liver insert and lower for the muscle (1.3 vs. 1.2). The CNR values for the other inserts also changed by approximately the same amount. These small changes do not change the conclusions drawn form the results.

The dose from CBCT with IBL is approximately 4 times lower than ortho‐pair dose and the very low dose volume (<0.1cGy) is less than half for the CBCT as compared to the ortho‐pair. The heterogeneity of the DVHs (Figs. 7–9) is due to the heterogeneity of target sites relative to the beam positions and the normal structures. A dose comparison study by Morin et al.^(21)^ and by Peng et al.^(22)^ showed a higher dose from the TBL CBCT than the ortho‐pair. In those studies, the authors used TBL for both CBCT and ortho‐pair with an exposure of 9 MU for the CBCT and either 4 or 5 MU for the ortho‐pair. Morin et al. also studied the possibility of fully incorporating the imaging dose into the treatment plan using a conventional TPS.

A direct comparison of image quality versus dose for other CBCT systems has not been conducted; however, several dosimetric comparisons have been undertaken. Using the Elekta system, Islam et al.^(23)^ reported 3.0 cGy and 1.6 cGy at the center of a head and body phantom, respectively. Using the Varian OBI system, Song et al.^(24)^ reported 8.5 cGy and 4.1 cGy at the center of the head and body phantom, respectively, and they reported similar dose results as Islam et al. for the Elekta system. The CNR as a function of dose will undoubtedly be higher for kV systems than either of the MV system; however, as Ding et al.^(25)^ point out in their Monte Carlo dosimetric study of kV CBCT images (in which they differentiated the dose to soft tissue and bones), the dose to bone can be 3 times higher than the dose to soft tissue. This is due to the predominant photoelectric effect of the kV photons in bone. This dose increase may be of consequence to a pediatric patient that has not yet reached growth maturity.

## V. CONCLUSIONS

The IBL CBCT improves image quality while simultaneously reducing the dose to the patient as compared to the TBL CBCT. A 1 cGy IBL CBCT, which can be used for bony anatomy localization, delivers a mean dose of less than one‐fourth as compared to conventional ortho‐pair films.

## ACKNOWLEDGEMENTS

Financial support provided by the American Lebanese Syrian Associated Charities (ALSAC) and Siemen OCS.
